# An Evaluation of Plasma TNF, VEGF-A, and IL-6 Determination as a Risk Marker of Atherosclerotic Vascular Damage in Early-Onset CAD Patients

**DOI:** 10.3390/jcm13061742

**Published:** 2024-03-18

**Authors:** Marta Bialecka, Michał Rac, Violetta Dziedziejko, Krzysztof Safranow, Dariusz Chlubek, Monika Ewa Rać

**Affiliations:** 1 Department of Internal Diseases and Hematology, Military Medical National Research Institute, Szaserów 128, 04-349 Warszawa, Poland; m.bialeckaaa@gmail.com; 2 Department of Diagnostic Imaging and Interventional Radiology, Pomeranian Medical University, Unii Lubelskiej 1, 71-252 Szczecin, Poland; hno15hno@gmail.com; 3 Department of Biochemistry, Pomeranian Medical University, Powstańców Wielkopolskich 72, 70-111 Szczecin, Poland; viola@pum.edu.pl (V.D.); chrissaf@mp.pl (K.S.); dchlubek@pum.edu.pl (D.C.)

**Keywords:** cytokines in plasma, coronary artery disease, plaque, intima–media, ankle-brachial index

## Abstract

**Background**: The pathogenesis of atherosclerosis is multifactorial and diverse. Pro-inflammatory cytokines are involved in these processes. It is suggested that inflammation may represent a novel and modifiable risk factor for cardiovascular disease. Therefore, this study aimed to gain insight into the relationship between plasma concentrations of TNF, VEGF, IL-6, and radiological parameters of atherosclerosis progression in patients with early-onset coronary artery disease (CAD). **Methods:** Seventy clinically stable patients were included in the study group. The age range for men was no more than 50 years, while for women, it was no more than 55 years. Fasting blood samples were obtained for plasma TNF, VEGF, and IL-6 protein measurements. Plasma cytokine concentrations were measured via ELISA. Doppler ultrasound of the carotid and peripheral arteries was performed in all patients. **Results:** After Bonferroni correction, there were no significant correlations between any cytokine and radiological parameters of atherosclerosis progression in our patients. **Conclusions:** The determination of plasma TNF, IL-6, and VEGF levels may not be a reliable marker for the vascular condition, and the measurement of these cytokines in plasma cannot replace the classical radiological examination of the vessels.

## 1. Introduction

Coronary artery disease (CAD) is a major challenge for public health. CAD is associated with high morbidity and high mortality worldwide but particularly in the Western world [1]. Usually, the disease affects older people, but the incidence of CAD has increased in recent decades in younger people. This CAD manifestation at a young age is known as early or premature CAD. In studies of early-onset CAD, the upper age limit is between 35 and 55 years [2]. In the era of preventive cardiology, a better understanding of this group of patients is important. Years before any clinical symptoms appear, the presence of risk factors for cardiovascular disease can be a threat for the future. This effectively complicates the management of clinicians, including cardiologists. The pathogenesis of atherosclerosis is multifactorial and diverse. It is associated with the activation of several signaling pathways through inflammatory or mechanical damage to the vascular endothelium, mobilizing the body’s immune processes. Pro-inflammatory cytokines are also involved in these processes, and inflammation is increasingly recognized as a new modifiable cardiovascular risk factor [3]. Primary and secondary prevention plays a key role in the avoidance of coronary events. In the pathophysiology of early-onset CAD, oxidative stress markers are important for endothelial dysfunction and inflammation. Understanding the pathways involved may help to define some aspects of vascular biology and additional risk markers.

Tumor necrosis factor (TNF) is a cachexin produced mainly by activated monocytes and macrophages. Smaller amounts of TNF are produced by endothelial cells, adipocytes, mast cells, T lymphocytes, and smooth muscle cells [4]. Human TNF is a glycoprotein in shorter or longer isoforms produced by tissues through the post-translational modification of a 212-amino-acid polypeptide. It is normally composed of 182 amino acids [5] and is a product of the TNF gene, which is located on chromosome 6 at position p21.3 and contains 4 exons. TNF affects cells by binding to the appropriate receptor (TNF-R1 or TNF-R2.) on the cell membrane surface. Stimulation of these receptors leads to the activation of cytokine production and realize the initiation of the arachidonic acid cascade. It leads to an increase in intracellular free radical concentration and also stimulates the liver to produce acute-phase proteins and adipose tissue to produce adiponectin [6]. TNF can affect the IL-6 secretion levels. TNF is a pro-atherosclerotic factor, but the mechanism is not fully understood [7]. For instance, there is still insufficient information regarding the impact of TNF on vascular damage in individuals diagnosed with coronary artery disease at a young age.

Interleukin 6 (IL-6) is a multifunctional pro-inflammatory cytokine. Its levels are also increased in atherosclerosis and acute ischemic conditions. It is produced by macrophages, T lymphocytes, and adipocytes in response to elevated levels of IL-1, TNF-α, interferon, viruses, and bacterial lipopolysaccharides [8]. IL-6 acts through its membrane-bound receptors (IL-6R) on monocytes, macrophages, neutrophils, lymphocytes, and hepatocytes. It also binds its blood-soluble receptors (sIL-6R) [9], and when bound in the IL-6/sIL-6R complex, it activates cells in the same way as interleukin itself. This cytokine is a mediator of the acute-phase response. IL-6 stimulates the liver to produce acute-phase proteins, such as fibrinogen, C-reactive protein (CRP), and plasma amyloid A (SAA). In the acute-phase response, the platelet count and activity increase, blood viscosity increases, and increased SAA decreases the HDL levels. Paracrine and autocrine activation of monocytes in the vessel wall by IL-6 contributes to fibrinogen deposition. IL-6 decreases plasma lipoprotein lipase activity, which increases the lipid uptake by macrophages. This leads to foam cell formation [10]. Circulating IL-6 affects the endocrine system. This cytokine stimulates the hypothalamic–pituitary–adrenal axis, the activation of which is associated with insulin resistance, hypertension, and central obesity [11]. Therefore, IL-6 is a proatherogenic cytokine and an important mediator of atherosclerotic disease or acute ischemic conditions [12,13]. The role of IL-6 in younger patients with CAD is less well understood. This cytokine may be a promising non-invasive marker for monitoring early CAD patients before and after acute coronary syndrome.

The vascular endothelial growth factor (VEGF-A) is a cytokine that belongs to the large family of vascular endothelial growth factors. VEGF plays a central role in the formation and function of blood vessels. It is a protein that promotes increased vascular permeability and monocyte migration across endothelial layers [14]. The molecular mechanism regulating angiogenesis in atherosclerosis involves the activation of signaling pathways by hypoxia and an increase in VEGF-A release. Endothelial cells in blood vessels close to the surface are supplied with VEGF-A by a positive gradient of VEGF-A. This condition promotes the uptake of oxLDL by macrophages and the survival of monocytes and macrophages [15]. The development and progression of atherosclerotic plaques is characterized by pathological angiogenesis of the vessel wall. VEGF is, therefore, a mitogen for endothelial cells, increasing their activity and proliferation rate. The biological effects of this cytokine are mediated by VEGFR1 and VEGFR2 receptors, which are expressed on the surface of vascular endothelial cells, monocytes, macrophages, platelets, megakaryocytes, hematopoietic cells, retinal stem cells, and osteoblasts [16]. In patients following acute myocardial ischemia, an increase in plasma VEGF-A levels promotes neovascularization but is also a trigger mechanism for the atherogenic process and atherosclerotic plaque formation [15]. It has been noted [17] that VEGF levels are elevated in the stable phase after Mi and correlate with inflammatory cytokines. In summary, VEGF stimulates neovascularization. An atherosclerotic plaque requires a blood supply. Chemotactic factors, such as tissue hypoxia, stimulate the release of VEGF proteins, leading to angiogenesis in the vessel wall. These developments often precede luminal plaque formation. VEGF levels may have an important negative prognostic value, because VEGF may provide a potential link to plaque destabilization, as one of many possible markers.

Unstable plaques are part of the widespread atherosclerotic process. An atherosclerotic process in the carotid region is associated with a higher incidence of CAD [18]. An ultrasonographic score based on the assessment of the carotid and femoral plaque burden predicts CAD more accurately than other non-radiographic methods. It is also a suitable non-invasive and safe method for detecting high-risk patients [19]. The IMC-T value > 0.6 mm was found to be a sensitive and specific ultrasound parameter that may be useful in detecting the presence of CAD [20]. The ankle-brachial index (ABI), as the ratio of ankle to arm systolic blood pressure, is a simple diagnostic test, also available in any primary care setting. An ABI < 0.9 is a diagnostic criterion for peripheral artery disease and a marker of future cardiovascular risk [21]. Ankle-brachial index (ABI) screening is useful in diagnosing peripheral artery disease with good sensitivity (75%) and specificity (86%) in high-risk patients with CAD [22]. The measurement of cytokines in peripheral blood is also a useful tool for assessing inflammatory responses in a variety of diseases. Immunoassays have advantages and disadvantages; however, they are generally known to be robust and reproducible. They are notably useful in clinical laboratories for measuring cytokines, as they are more widely available than an examination by a specialist radiologist. Therefore, this study aimed to gain insights into the relationship between plasma concentrations of TNF, VEGF, and IL-6 and radiological parameters of atherosclerotic progression in patients with early CAD. We wanted to test the hypothesis that the measurement of some cytokines in plasma could replace the classic radiological examination of the vessels.

## 2. Materials and Methods

This study included 70 clinically stable patients with early-onset CAD, comprising 18 women and 52 men (aged 50 years or less in men and 55 years in women). Age was an inclusion criterion for this study, as only people with strong risk factors develop early-onset CAD. The criteria for the CAD diagnosis were one of three conditions: a history of myocardial revascularization surgery (CABG or PTCA) or angiographically documented coronary artery stenosis (≥50% of the left main stem coronary artery or ≥70% of the branches) or a history of myocardial infarction. To be included in this study, each patient had to have undergone a potential revascularization procedure or coronary angiography at least 30 days prior. Patients with acute coronary syndrome, significant heart failure or heart disease (NYHA ≥ II), liver failure or severe kidney disease (serum creatinine > 3 mg/dL), cancer, type 1 diabetes, thyroid disease, or rheumatoid arthritis were excluded from the study. The patients were all hospitalized at the Department of Cardiology, Szczecin District Hospital, Szczecin, Poland. Table 1 details past cardiac procedures, treatments used, risk factors, as well as the clinical characteristics of the CAD group included in the study. The biochemical control group comprised 50 healthy individuals without CAD and was selected based on the same exclusion criteria as the study group. Additionally, the control group was matched to the study group in terms of age and sex (74% male; mean 48 ± 3.20 years). The control group was randomly selected from persons attending regular medical check-ups at the occupational health clinic. The study was approved by our institutional ethics committee (BN-001/162/04) and adhered to the tenets of the Declaration of Helsinki. All subjects gave their informed consent for inclusion in the study before their participation in the study.

Fasting blood samples were taken for plasma TNF, VEGF, and IL-6 protein measurements. Plasma samples were stored at −80 °C until analysis. Plasma cytokine concentrations were measured using commercially available enzyme-linked immunosorbent assay (ELISA) kits (EIAab, Wuhan EIAab Science Co., Ltd., Wuhan, China) according to the manufacturer’s protocol, as previously published by Bialecka et al. [23]. An ELX 808IU automated microplate reader (Bio-Tek Instruments Inc., Winooski, VT, USA), adequately calibrated with recombinant human protein concentrations in the appropriate range, was used to determine the plasma concentration of the tested proteins. Calibration was performed with recombinant human TNF in a concentration range of 0.5–32 pg/mL. The absorbance of the test samples for TNF measurement was read against the calibration curve at 490 nm. The assays were evaluated, and the minimum detectable dose (MDD) of TNF ranged from 0.038 to 0.191 pg/mL. The mean MDD was 0.106 pg/mL. The MDD was determined by adding two standard deviations to the mean optical density value of twenty standard replicates and calculating the corresponding concentration. The intra-assay precision (CV%) was 3.1–8.5%, and the inter-assay precision was 7.3–10.6%. Calibration was performed with recombinant human IL-6 in a concentration range 0.156–10 pg/mL. The absorbance of the test samples for IL-6 measurement was read at 490 nm. The detection limit was 0.039 pg/mL. The intra-assay precision (CV%) was 5.5–9.8%, and the inter-assay precision was 5.5–11.2%. Calibration was performed with recombinant human VEGF in a concentration range 31.3–2000 pg/mL. The absorbance of the VEGF test samples was read at 450 nm. The detection limit was 9 pg/mL. The intra-assay precision (CV%) was 4.5–6.7%, and the inter-assay precision was 6.2–8.8%.

Doppler ultrasound of the carotid and peripheral arteries was performed in all patients. The examinations were conducted by a highly experienced radiologist. Common carotid artery (CCA) and brachial artery intima–media complex (IMC) thickness, density, and atheromatous plaque thickness at the CCA bifurcation were measured using the M`Ath program [24,25]. Density measurement was based on the intensity of reflection from the plaque, which is dependent on calcium deposits. The ankle-brachial index (ABI) was calculated by dividing the systolic blood pressure in the arteries of the left and right ankle (posterior tibial artery) or foot (anterior tibial artery) by the higher of the two systolic blood pressures in the arms.

The distributions of the measurable clinical parameters were significantly different from the usual ones (the Shapiro–Wilk test); thus, in the calculations, we used non-parametric tests, including, first, the Kruskal–Wallis test, followed by the Mann–Whitney U test. The non-parametric Mann–Whitney U test was used in the calculations for gender differences in cytokine levels and the radiological parameters studied. Correlations between quantitative variables and cytokine plasma concentrations were assessed using Spearman’s rank correlation coefficient (Rs). *p*-values < 0.05 were considered statistically significant, but the classical Bonferroni correction was also applied in the significance analysis. As a total of 18 statistical associations (including correlations and comparisons in the whole group and the subgroups) were analyzed in this study, we applied the Bonferroni-corrected threshold *p*-value equal to 0.05/18 = 0.0028. Statistical calculations were performed with Statistica 6.1 software.

## 3. Results

The plasma levels of the tested cytokines are shown in Table 2. For further analysis, CAD cases were divided into subgroups of women and men. In our study, plasma TNF levels were not significantly different (*p* = 0.052) but were lower in CAD patients (1.33 ± 0.36 pg/mL) than in controls (1.51 ± 0.005 pg/mL). No statistically significant differences in plasma TNF, IL-6, and VEGF concentrations were observed between female and male groups. No statistically significant difference (*p* = 0.19) was observed in plasma IL-6 levels between the study (1.68 ± 2.74 pg/mL) and control (1.47 ± 0.33 pg/mL) groups, but IL-6 levels were higher in the CAD group. Similarly, no statistically significant difference (*p* = 0.53) was observed in plasma IL-6 levels between the study group (236 ± 17.2 pg/mL) and the control group (220 ± 77.5 pg/mL), but VEGF levels were higher in the CAD group. Gender differences in the studied radiological parameters were compared and are presented in Table 3. The Bonferroni correction for multiple comparisons was applied to the significance analysis, with a resulting value of 0.0028. In the classical calculation, statistically significant differences were observed between the female and male groups in some radiological parameters, such as ankle-brachial index on the right and left side and in the mean value. There were also statistically significant differences between female and male groups in the intima–media complex of the common carotid arteries on the left side and in the mean value. Similarly, statistically significant differences were observed between female and male groups in the intima–media complex of brachial arteries on the right and left side and in mean value. There were no significant associations between gender and any of the plaque parameters of the common carotid arteries and bifurcation, except for mean plaque thickness. After applying the Bonferroni correction, it was found that only the association between gender and the intima–media complex of brachial arteries on the left side remained significant among all the parameters analyzed. Except for this one, none of the analyzed parameters remained statistically significant after applying the Bonferroni correction.

Correlations between cytokine concentrations are shown in Table 4. In the classical calculation, a statistically significant correlation was found between IL-6 and VEGF plasma levels, but upon applying the Bonferroni correction, the statistical significance of this correlation was found to be non-existent. Correlations between cytokine plasma concentrations and quantitative variables were assessed using Spearman’s rank correlation coefficient and are presented in Table 5. In the classical calculation, statistically significant correlations were found but only in terms of borderline significance, very weak correlation with higher plaque density on the right side (*p* = 0.083) value in patients with higher TNF plasma concentrations, and lower intima–media complex of the brachial artery with higher IL-6 plasma concentrations (*p* = 0.07). We also observed only a slightly negative correlation of IL-6 with the intima–media complex of the left brachial artery (*p* = 0.02). Nonetheless, after the application of the Bonferroni correction, none of the analyzed parameters were found to be statistically significant. There were no significant correlations between VEGF plasma concentrations and any of the radiological parameters. Finally, there were no significant correlations between any of the cytokines and radiological parameters of atherosclerosis progression in our patients after Bonferroni correction. Moreover, patients were categorized into subgroups, with abnormal vs. normal vascular parameters values based on the presence or absence of plaque, and ABI < 0.9, IMC cca > 0.9, and imc ba > 0.6 values, following the clinical guidelines from other authors [26,27,28,29,30]. The association between the concentration of the cytokines studied and vascular abnormalities was investigated. The Mann–Whitney U test indicated only one slight significance (*p* = 0.031) in TNF levels when comparing cases with IMC cca > 0.9 and IMC cca < 0.9. However, after applying the Bonferroni correction, no statistical significance was observed.

## 4. Discussion

Intima–media thickness (IMT) measured in peripheral arteries correlates with the presence and progression of atherosclerosis in the coronary arteries. IMT measurements can help to select high-risk patients and assess the effectiveness of therapy. Patients with higher IMT values have been reported to have a higher incidence of cardiac events. Ultrasound assessment of the IMT complex of peripheral arteries in daily clinical practice helps monitor the efficacy of pharmacological therapy in CAD patients after MI [26]. The ankle-brachial index (ABI) is an independent prognostic marker for cardiovascular events in patients with coronary artery disease (CAD). Routine ABI screening may have important prognostic value in these patients [27]. The range for a normal ankle-brachial index (ABI) is considered to be between 0.9 and 1.4. Abnormal ABI is independently associated with all-cause mortality (RR: 1.74; 95% CI: 1.32–2.30) in patients with CAD, even after adjustment for conventional confounders. However, the prognostic value of an abnormal ABI is dominated by a low rather than a high ABI [28]. This simple and cost-effective research could serve as a crucial prognostic tool for CAD patients and may help in further classifying extremely high-risk CAD patients who may require more intensive monitoring or treatment [29]. The higher risk of atherosclerosis is associated with lower ABI and higher IMC thickness [30]. Plaque density reflects plaque calcification. Lower plaque calcification is associated with plaque instability, whereas high plaque calcification is associated with a predisposition to thrombosis [31]. Inflammation plays a fundamental role in the process of atherosclerosis [32]. Therefore, we investigated the correlation between radiological factors of the atheromatous plaque and plasma levels of three proatherogenic cytokines: TNF, IL-6, and VEGF. We tested the hypothesis that the measurement of plasma levels of some cytokines may be a marker of cardiovascular risk in cases of early coronary heart disease. We found no significant associations between plasma TNF, IL-6, and VEGF concentrations and any of the parameters measured by Doppler ultrasound of the carotid and peripheral arteries.

Other authors have reported different TNF plasma concentrations. The range is from a few [33] to several tens of pg/mL [34] in CAD patients, while in healthy people, it is about 2 pg/mL [35]. Our patients were all treated with statins, which reduce plasma TNF levels [36]. In contrast, there are no precise reference ranges for IL-6 in the available literature. Todd et al. [37] found a mean value of 1.89 pg/mL for plasma from healthy subjects. Similar results were found by Hennø et al. [38], suggesting that the IL-6 levels measured in our early CAD cases were normal. It should be noted that an IL-6 level above 1 pg/mL is considered to be highly predictive in CAD patients with intermediate atherosclerotic cardiovascular risk and chest pain [39]. Other authors have reported reductions in plasma VEGF concentrations in patients taking statins [40,41]. In the cited studies, plasma VEGF concentrations were higher in CAD cases than in controls before statin treatment and decreased after two months of statin therapy. In our study, all cases received statins (mainly atorvastatinum or simvastatinum at a daily dose of 20–40 mg). This is a treatment for lowering LDL cholesterol, but it also has pleiotropic effects on endothelial function [42] and may have anti-inflammatory effects [43]. In addition, the authors of a meta-analysis study reported that the range of plasma VEGF levels in healthy subjects is 51–391 pg/mL [44]. The mean plasma VEGF levels reported in our study were not high, ranging from 31.4 to 938.1 pg/mL, but were similar to those reported in studies by other authors [45,46,47].

There is a limited amount of research that has focused on the link between cytokines and Doppler examination’s comprehensive measurements of atherosclerotic vascular lesions. Elkind et al. reported that serum-soluble TNF receptor concentrations were significantly associated with carotid plaque thickness in a community study population aged between 40 and 70 years [48]. In another study, TNF receptor expression on monocytes was positively associated with carotid intima–media thickness in healthy women aged 20–50 years [49]. In a study by Andersson et al. [50], plaque size was associated with increased plasma TNF levels in subjects aged 70 years. In elderly patients, the length, thickness, and number of carotid atherosclerotic plaques with plasma TNF levels decreased after statin treatment [51]. On the other hand, in a GWAS study, no SNP in the TNF gene was associated with carotid intima–media thickness in hypertensive subjects from British Caucasian families [52]. Furthermore, carotid intima–media thickness was not associated with plasma TNF levels in patients with manifest cardiovascular diseases [53]. This observation is consistent with our study. Based on our study, we observed only a borderline significance and a very weak correlation between higher plasma TNF levels and increased plaque density in the right common carotid artery of the patients.

In other studies, IL-6 levels correlate positively with the degree of carotid stenosis, the presence of unstable plaque, and the dynamics of changes in the morphology of atherosclerotic plaques in the internal carotid artery [54,55]. The authors do not agree on whether there is an association between IL-6 and carotid IMC thickness, a parameter used to detect and monitor early atherosclerotic lesions [56]. However, associations between IL-6 levels and IMC thickness of the carotid bulb and internal carotid artery have been demonstrated, though these results have not been confirmed by other studies after adjustment for conventional risk factors [57]. This observation is also consistent with our study. We found only a borderline significant correlation of the lower brachial artery intima–media complex with higher plasma IL-6 concentrations. We also observed only a slight and very weak negative correlation of IL-6 with the intima–media complex of the left brachial artery. However, none of these analyzed parameters remained statistically significant after applying the Bonferroni correction.

Only one study [58] found a significant correlation between mean carotid IMT and VEGF levels in Behçet’s disease cases (r = 0.317, *p* < 0.05). Another study [59] failed to confirm a change in plasma VEGF levels and anatomical location of the coronary artery, aortic root, coronary sinus, and femoral vein immediately after angioplasty in CAD cases. Similarly, in another study [60], there was no significant correlation between plasma VEGF levels and carotid intima–media thickness in post-acute thrombotic stroke cases 90 days after stroke onset. The authors concluded that plasma VEGF cannot specifically assess vascular status because it can be induced by ischemic conditions in tissues throughout the body. This observation is also consistent with our study. There were no significant correlations between plasma VEGF concentrations and any of the radiological parameters.

This study is subject to several limitations that should be noted. One limitation is the absence of a randomly selected control group comprising healthy individuals without CAD, who would have satisfied the same exclusion criteria as the study group, including age and sex. Although a biochemical control group was included in the study, the lack of a corresponding control group for radiological studies is another limitation that should be acknowledged in future research. Another limitation of this study is that one of the major challenges in cytokine analysis is the availability of a suitable analytical tool with high accuracy, specificity, precision, stability, linearity, and analytical sensitivity. In addition, the measurement of cytokines in human blood has not yet been properly calibrated. It is important to acknowledge that in experimental research, it is nearly impossible to eliminate the influence of all potential confounding factors. Despite this challenge, we made a concerted effort to thoughtfully consider the inclusion and exclusion criteria for the trials to mitigate their impact as much as possible. However, any unrecognized inflammation before the study, anti-inflammatory therapy applied by the patient (without the doctor’s knowledge), or undiagnosed cancer and diabetes can significantly affect the levels of cytokines tested.

There have been major advances in our understanding of the epidemiology and pathophysiology of multidisciplinary atherosclerotic disease, but little is known about the association between carotid artery disease and peripheral arterial disease. Carotid artery stenosis is associated with the presence of hypoechoic unstable plaques. These plaques are associated with high levels of inflammatory markers and present a large infiltration of macrophages. The greater prevalence of hypoechoic unstable carotid plaques could explain why peripheral arterial disease is associated with a higher risk of stroke than coronary artery disease [61]. The role of inflammation in atherothrombotic disease is being increasingly recognized. Several biomarkers of inflammation are associated with ultrasonographic measures of carotid artery atherosclerosis in patients with moderate to high prevalence of CAD. These include markers of hemostasis (fibrinogen and selectin), inflammatory variables (hsCRP, calprotectin and SAA), cytokines (TNF, MCP-1, IL-6 and IL-8), and cell adhesion molecules (VCAM-1 and ICAM-1) [53,62,63,64]. In addition, different classes of cytokines (chemokines, TNF family, IL1 family, interferons, and adipokines) are implicated in the entire process, leading to the destabilization of the atherosclerotic plaque and, consequently, to the incidence of myocardial infarction. In particular, the TNF and IL1 families are involved in inflammatory cell accumulation, platelet aggregation, vulnerable plaque formation, cardiomyocyte apoptosis, and adverse remodeling after myocardial infarction [3]. Compared to stable CAD and healthy subjects, acute coronary syndromes are associated with long-term increases in serum concentrations of anti- and pro-inflammatory cytokines, such as IL-2, IL-10, and TNF. It seems likely that sudden CAD progression leading to acute coronary syndromes is triggered/accompanied by prolonged immune activation [65].

Considering the exhaustive nature of this study on proinflammatory cytokines and early-onset coronary artery disease (CAD), it seems important that future research should focus on the potential role of patient demographics and lifestyle factors in modulating cytokine levels and their consequent impact on CAD. To gain a better understanding of the relationship between cytokines and atherosclerotic changes, research could be undertaken considering additional variables, such as age, gender, dietary habits, and physical activity levels. In addition, considering the variability in cytokine expression due to genetic factors may provide a more nuanced understanding of their role in CAD pathology.

## 5. Conclusions

Although there has been extensive research on the involvement of inflammatory parameters in the pathogenesis of CAD, our study revealed a lack of association between certain cytokine concentrations and the radiological parameters of vessels. These observations are consistent with other studies. While immunoassays are a suitable method for measuring cytokines in clinical laboratories and are much more readily available than an examination by a specialist radiologist, it appears that the determination of plasma TNF, IL-6, and VEGF levels cannot be a surrogate test for vascular status. Therefore, it is necessary to note that measuring cytokines in plasma is not a substitute for the traditional radiological examination of the vessels.

## Figures and Tables

**Table 1:** Study group characteristics (number of cases—70).

Parameter	Value
Gender (number of males)	52
Patients age (years)	49.9 ± 5.91
BMI (kg/m^2^)	28.4 ± 4.2
Past MI (number of cases)	49
Age of the first MI (years)	44.0 ± 5.6
Time since diagnosis of MI to joining the program (years)	3.20 ± 0.74
History of hypertension (number of cases)	46
Age at diagnosis of hypertension (years)	42.6 ± 8.6
Systolic BP (mmHg)	128 ± 15.0
Diastolic BP (mmHg)	77.6 ± 8.6
Past PTCA (number of cases)	50
Past CABG (number of cases)	26
Past smoking (number of cases)	62
Years smoking	18.9 ± 9.8
Current smoking	8
Diabetes type 2 (number of cases)	9
Statins (number of cases)	70
Anti-platelet drugs (Aspirin, number of cases)	63
ACEI (number of cases)	56
Beta-blockers (number of cases)	62
Diuretics (number of cases)	22
ARB (number of cases)	12
Calcium channel blockers (number of cases)	13

BMI = body mass index, MI = myocardial infarction, BP = blood pressure, PTCA = percutaneous transluminal coronary angioplasty, CABG = coronary artery bypass grafting, ACEI = angiotensin 1 converting enzyme inhibitors, ARB = angiotensin 2 receptor blockers.

**Table 2:** Plasma levels of the tested cytokines (the whole CAD group and subgroups).

Parameter	Study Group	F ^1^	M ^2^	*p*-Value
TNF (pg/mL)	1.33 ± 0.36	1.69 ± 1.21	1.37 ± 0.37	0.15
IL-6 (pg/mL)	1.68 ± 2.74	1.69 ± 1.21	1.73 ± 3.18	0.10
VEGF (pg/mL)	236 ± 17.2	236 ± 22.3	213 ± 26.2	0.12

^1^ the subgroup of women, ^2^ the subgroup of men.

**Table 3:** Gender differences in the studied radiological parameters.

Parameter	*p*-Value
ABI right	0.01
ABI left	0.04
ABI mean	0.004
IMC cca right	0.18
IMC cca left	0.07
IMC cca mean	0.04
IMC ba right	0.08
IMC ba left	0.0021
IMC ba mean	0.020
PLA thickness left	0.24
PLA length left	0.30
PLA density left	0.58
PLA thickness right	0.63
PLA length right	0.85
PLA density right	0.37
PLA thickness mean	0.04
PLA length mean	0.97
PLA density mean	0.88

ABI—ankle-brachial index, IMC cca—intima–media complex of common carotid arteries, PLA—plaque of common carotid arteries and bifurcation, IMC ba—intima–media complex of brachial arteries, mean—value calculated as mean of measurements of the right and left arteries.

**Table 4:** Correlations between cytokine concentration.

Cytokines	Rs	*p*-Value
IL-6 & VEGF	0.20	0.05
IL-6 & TNF	0.17	0.10
TNF& VEGF	0.10	0.35

**Table 5:** Correlations between cytokine concentration and quantitative parameters in the whole CAD group.

Parameter	Correlations with TNF		Correlations with IL-6		Correlations with VEGF	
	Rs	*p*-Value	Rs	*p*-Value	Rs	*p*-Value
ABI right	−0.08	0.51	−0.12	0.32	−0.12	0.35
ABI left	−0.08	0.52	−0.10	0.40	−0.16	0.19
ABI mean	−0.07	0.56	−0.12	0.32	−0.15	0.23
IMC cca right	0.01	0.91	−0.04	0.73	−0.04	0.74
IMC cca left	0.06	0.65	0.02	0.86	0.01	0.91
IMC cca mean	0.07	0.56	−0.03	0.80	−0.04	0.74
IMC ba right	−0.12	0.32	−0.12	0.32	−0.16	0.19
IMC ba left	−0.001	1.00	−0.30	0.02	−0.16	0.19
IMC ba mean	−0.12	0.33	−0.22	0.07	−0.10	0.41
PLA thickness left	0.06	0.76	−0.17	0.42	−0.19	0.35
PLA length left	−0.07	0.73	0.22	0.28	−0.03	0.88
PLA density left	−0.31	0.12	0.01	0.97	−0.15	0.46
PLA thickness right	0.08	0.66	−0.12	0.52	0.27	0.12
PLA length right	−0.16	0.38	0.01	0.96	0.10	0.58
PLA density right	0.30	0.083	−0.02	0.92	−0.01	0.94
PLA thickness mean	−0.12	0.50	−0.10	0.59	0.24	0.17
PLA length mean	−0.02	0.89	0.04	0.79	0.09	0.57
PLA density mean	0.08	0.63	0.03	0.86	−0.03	0.86

## Data Availability

The research data are available from the Department of Biochemistry, Pomeranian Medical University.

## References

[1] (2018). Statistical Yearbook of the Republic of Poland 2018.

[2] Aggarwal A., Srivastava S., Velmurugan M. (2016). Newer perspectives of coronary artery disease in young. World J. Cardiol..

[3] Mourouzis K., Oikonomou E., Siasos G., Tsalamadris S., Vogiatzi G., Antonopoulos A., Fountoulakis P., Goliopoulou P., Papaioannou S., Tousoulis D. (2020). Pro-inflammatory Cytokines in Acute Coronary Syndromes. Curr. Pharm..

[4] Wallach D. (2016). The cybernetics of TNF: Old views and newer ones. Semin. Cell Dev. Biol..

[5] Horiuchi T., Mitoma H., Harashima S.-I., Tsukamoto H., Shimoda T. (2010). Transmembrane TNF-alpha: Structure, function and interaction with anti-TNF agents. Rheumatology.

[6] Akash M.S.H., Rehman K., Liaqat A. (2018). Tumor Necrosis Factor-Alpha: Role in Development of Insulin Resistance and Pathogenesis of Type 2 Diabetes Mellitus. J. Cell Biochem..

[7] Popko K., Gorska E., Stelmaszczyk-Emmel A., Plywaczewski R., Stoklosa A., Gorecka D., Pyrzak B., Demkow U. (2010). Proinflammatory cytokines IL-6 and TNF-α and the development of inflammation in obese subjects. Eur. J. Med. Res..

[8] Reiss A.B., Siegart N.M., De Leon J. (2017). Interleukin-6 in atherosclerosis: Atherogenic or atheroprotective?. Clin. Lipidol..

[9] Bacchiega B.C., Bacchiega A.B., Usnayo M.J., Bedirian R., Singh G., da Rocha G., Pinheiro C. (2017). Interleukin 6 inhibition and coronary artery disease in a high-risk population: A prospective community-based clinical study. J. Am. Heart Assoc..

[10] Schaper F., Rose-John S. (2015). Interleukin-6: Biology, signaling and strategies of blockade. Cytokine Growth Factor Rev..

[11] Yudkin J.S., Kumari M., Humphries S.E., Mohamed-Ali V. (2000). Inflammation, obesity, stress and coronary heart disease: Is interleukin-6 the link?. Atherosclerosis.

[12] Su D., Li Z., Li X., Chen Y., Zhang Y., Ding D., Deng X., Xia M., Qiu J., Ling W. (2013). Association between serum interleukin-6 concentration and mortality in patients with coronary artery disease. Mediat. Inflamm..

[13] Clarke R., Valdes-Marquez E., Hill M., Gordon J., Farrall M., Hamsten A., Watkins H., Hopewell J.C. (2018). Plasma cytokines and risk of coronary heart disease in the PROCARDIS study. Open Heart.

[14] Koczy-Baron E., Kasperska-Zając A. (2014). Rola naczyniowo-śródbłonkowego czynnika wzrostu w procesach zapalnych. Postep. Hig. Med. Dosw..

[15] Ríos-Navarro C., Hueso L., Díaz A., Marcos-Garcés V., Bonanad C., Ruiz-Sauri A., Vila J.M., Sanz M.J., Chorro F.J., Piqueras L. (2021). Role of antiangiogenic VEGF-A165b in angiogenesis and systolic function after reperfused myocardial infarction. Rev. Esp. Cardiol. (Engl. Ed.).

[16] Ferrara N., Gerber H.P., le Couter J. (2003). The biology of VEGF and its receptors. Nat. Med..

[17] Erżen B., Šilar M., Šabovič M. (2014). Stable phase post-Mi patients have elevated VEGF levels correlated with inflammation markers, but not with atherosclerotic burden. BMC Cardiovasc. Disord..

[18] Drzewiecka-Gerber A., Rybicka-Musialik A., Myszor J., Ziaja D., Trusz-Gluza M. (2012). Impact of atherosclerotic changes of carotid vessels on long-term outcome in relatively young patients with acute coronary syndrome. Kardiol. Pol..

[19] Koulouri A., Darioli R., Qanadli S.D., Katz E., Eeckhout E., Mazzolai L., Depairon M. (2021). The atherosclerosis burden score. Vasa.

[20] Kłosiewicz-Wasek B., Ceremuzyński L., Poloński L., Lukaszewicz R., Wasilewski J. (2008). Association between carotid artery atherosclerosis and coronary artery disease in young females. Reference to sex hormone profile. Kardiol. Pol..

[21] Ahn S., Jo E.A., Min S.K., Min S., Ha J., Park K.W., Min K.B. (2020). Predictive Value of Abnormal and Borderline Ankle-Brachial Index for Coronary Re-Intervention and Mortality in Patients with Coronary Artery Disease: An Observational Cohort Study. Vasc. Spec. Int..

[22] Minami H.R., Itoga N.K., George E.L., Garcia-Toca M. (2021). Cost-effectiveness analysis of ankle-brachial index screening in patients with coronary artery disease to optimize medical management. J. Vasc. Surg..

[23] Białecka M., Dziedziejko V., Safranow K., Krzystolik A., Marcinowska Z., Chlubek D., Rać M.E. (2024). Could TNF be a metabolic syndrome and left ventricular hypertrophy marker in patients with early onset coronary artery disease?. Diagnostics.

[24] Touboul P.J., Hennerici M.G., Meairs S., Adams H., Amarenco P., Bornstein N., Csiba L., Desvarieux M., Ebrahim S., Hernandez R. (2012). Mannheim carotid intima-media thickness and plaque consensus (2004–2006–2011). An update on behalf of the advisory board of the 3rd, 4th and 5th watching the risk symposia, at the 13th, 15th and 20th European Stroke Conferences, Mannheim, Germany, 2004, Brussels, Belgium, 2006, and Hamburg, Germany, 2011. Cerebrovasc. Dis..

[25] Iannuzzi A., Rubba P., Gentile M., Mallardo V., Calcaterra I., Bresciani A., Covetti G., Cuomo G., Merone P., Di Lorenzo A. (2021). Carotid Atherosclerosis, Ultrasound and Lipoproteins. Biomedicines.

[26] Lisowska A., Knapp M., Bolińska S., Lisowski P., Krajewska A., Sobkowicz B., Musiał W.J. (2012). The importance of intima-media thickness (IMT) measurements in monitoring of atherosclerosis progress after myocardial infarction. Adv. Med. Sci..

[27] Berkovitch A., Iakobishvili Z., Fuchs S., Atar S., Braver O., Eisen A., Glikson M., Beigel R., Matetzky S. (2022). Peripheral artery disease, abnormal ankle-brachial index, and prognosis in patients with acute coronary syndrome. Front. Cardiovasc. Med..

[28] Liu L., Sun H., Nie F., Hu X. (2020). Prognostic Value of Abnormal Ankle-Brachial Index in Patients With Coronary Artery Disease: A Meta-Analysis. Angiology.

[29] Hashizume N., Miura T., Miyashita Y., Motoki H., Ebisawa S., Izawa A. (2017). Prognostic value of ankle-brachial index in patients undergoing percutaneous coronary intervention: In- hospital and 1-year outcomes from the SHINANO Registry. Angiology.

[30] Weatherley B.D., Nelson J.J., Heiss G., Chambless L.E., Sharrett A.R., Nieto F.J., Folsom A.R., Rosamond W.D. (2007). The association of the ankle-brachial index with incident coronary heart disease: The Atherosclerosis Risk In Communities (ARIC) study, 1987–2001. BMC Cardiovasc. Disord..

[31] Alexopoulos N., Raggi P. (2009). Calcification in atherosclerosis. Nat. Rev. Cardiol..

[32] Bäck M., Yurdagul A., Tabas I., Öörni K., Kovanen P.T. (2019). Inflammation and its resolution in atherosclerosis: Mediators and therapeutic opportunities. Nat. Rev. Cardiol..

[33] Wu Y., Wang L., Zhan Y., Zhang Z., Chen D., Xiang Y., Xie C. (2022). The expression of SAH, IL-1β, Hcy, TNF-α and BDNF in coronary heart disease and its relationship with the severity of coronary stenosis. BMC Cardiovasc. Disord..

[34] Fang C., Chen Z., Zhang J., Pan J., Jin X., Yang M., Huang L. (2022). The value of serum YKL-40 and TNF-α in the diagnosis of acute ST-segment elevation myocardial infarction. Cardiol. Res. Pract..

[35] Wilczyński M., Krejca M., Stepinski P., Rozalski M., Golanski J. (2023). Platelet reactivity expressed as a novel platelet reactivity score is associated with higher inflammatory state after coronary artery bypass grafting. Arch. Med. Sci..

[36] Abbasifard M., Kandelouei T., Aslani S., Razi B., Imani D., Fasihi M., Cicero F.G., Sahebkar A. (2022). Effect of statins on the plasma/serum levels of inflammatory markers in patients with cardiovascular disease, a systematic review and meta-analysis of randomized clinical trials. Inflammopharmacology.

[37] Todd J., Simpson P., Estis J., Torres V., Wub A.H. (2013). Reference range and short- and long-term biological variation of interleukin (IL)-6, IL-17A and tissue necrosis factor-alpha using high sensitivity assays. Cytokine.

[38] Hennø L.T., Storjord E., Christiansen D., Bergseth G., Ludviksen J.K., Fure H., Barene S., Nielsen E.W., Mollnes T.E., Brekke O.L. (2017). Effect of the anticoagulant, storage time and temperature of blood samples on the concentrations of 27 multiplex assayed cytokines—Consequences for defining reference values in healthy humans. Cytokine.

[39] Wainstein M.V., Mossmann M., Araujo G.N., Gonçalves S.C., Gravina G.L., Sangalli M., Veadrigo F., Matte R., Reich R., Costa F.G. (2017). Elevated serum interleukin-6 is predictive of coronary artery disease in intermediate risk overweight patients referred for coronary angiography. Diabetol. Metab. Syndr..

[40] Dulak J., Loboda A., Jazwa A., Zagorska A., Dörler J.H. (2005). Atorvastatin affects several angiogenic mediators in human endothelial cells. Endothelium.

[41] Alber H.F., Dulak J., Frick M., Dichtl W., Schwarzacher S.P., Pachinger O. (2002). Atorvastatin decreases vascular endothelial growth factor in patients with coronary artery disease. J. Am. Coll. Cardiol..

[42] Barale C., Frascaroli C., Senkeev R., Cavalot F., Russo I. (2018). Simvastatin effects on inflammation and platelet activation markers in hypercholesterolemia. BioMed Res. Int..

[43] Schultz N.E., Hasseldam H., Rasmussen R.S., Vindegaard N., McWilliam O., Iversen H.K. (2019). Statin treatment before stroke reduces pro-inflammatory cytokine levels after stroke. Neurol. Res..

[44] Kut C., Mac Gabhann F., Popel A.S. (2007). Where is VEGF in the body? A meta-analysis of VEGF distribution in cancer. Br. J. Cancer.

[45] Kotschy M., Witkiewicz W., Grendziak R., Dubis J., Zapotoczny N., Kotschy D. (2012). Selected clotting factors in blood of patients with abdominal aortic aneurysms. Kardiol. Pol..

[46] Fei Y., Hou J., Xuan W., Zhang C., Meng X. (2018). The relationship of plasma mir-503 and coronary collateral circulation in patients with coronary artery disease. Life Sci..

[47] Danzig V., Míková B., Kuchynka P., Benáková H., Zima T., Kittnar O. (2010). Levels of circulating biomarkers at rest and after exercise in coronary artery disease patients. Physiol. Res..

[48] Elkind M.S., Cheng J., Boden-Albala B., Rundek T., Thomas J., Chen H., Rabbani R.E., Sacco L.R. (2002). Tumor necrosis factor receptor levels are associated with carotid atherosclerosis. Stroke.

[49] Cortez-Cooper M., Meaders E., Stallings J., Haddow S., Kraj B., Sloan G., McCully K.K., Cannon J.G. (2013). Soluble TNF and IL-6 receptors: Indicators of vascular health in women without cardiovascular disease. Vasc. Med..

[50] Andersson J., Sundström J., Kurland L., Gustavsson T., Hulthe J., Elmgren A., Zilmer K., Zilmer M., Lind L. (2009). The carotid artery plaque size and echogenicity are related to different cardiovascular risk factors in the elderly: The Prospective Investigation of the Vasculature in Uppsala Seniors (PIVUS) study. Lipids.

[51] Zhang H., Jiang M., Hou H., Li Q. (2021). Efficacy of simvastatin on carotid atherosclerotic plaque and its effects on serum inflammatory factors and cardiocerebrovascular events in elderly patients. Exp. Ther. Med..

[52] Cunnington M.S., Mayosi B.M., Hall D.H., Avery P.J., Farrall M., Vickers M.A., Watkins H., Keavney B. (2009). Novel genetic variants linked to coronary artery disease by genome-wide association are not associated with carotid artery intima-media thickness or intermediate risk phenotypes. Atherosclerosis.

[53] Larsson P.T., Hallerstam S., Rosfors S., Wallén N.H. (2005). Circulating markers of inflammation are related to carotid artery atherosclerosis. Int. Angiol..

[54] Puz P., Lasek-Bal A. (2017). Repeated measurements of serum concentrations of TNF-alpha, interleukin-6 and interleukin-10 in the evaluation of internal carotid artery stenosis progression. Atherosclerosis.

[55] Okazaki S., Sakaguchi M., Miwa K., Furukado S., Yamagami H., Yagita Y., Mochizuki H., Kitagawa K. (2014). Association of interleukin-6 with the progression of carotid atherosclerosis: A 9-year follow-up study. Stroke.

[56] Huang Y.Q., Li J., Chen J.Y., Zhou Y.L., Cai A.P., Huang C., Feng Y.Q. (2017). The Association of Circulating MiR-29b and Interleukin-6 with Subclinical Atherosclerosis. Cell Physiol. Biochem..

[57] Tamariz L., Hare J.M. (2010). Inflammatory cytokines in heart failure: Roles in aetiology and utility as biomarkers. Eur. Heart J..

[58] Öztürk M., Ünverdi S., Oktar S., Bukan N., Gülbahar O., Ureten K., Göker B., Haznedaroglu S., Sungur G., Ciftçi T.U. (2008). Vascular endothelial growth factor and carotid intima-media thickness in patients with Behçet’s disease. Clin. Rheumatol..

[59] Jaumdally R., Varma C., Blann A., MacFadyen R.J., Gregory Y.H. (2007). Systemic and intracardiac vascular endothelial growth factor and angiopoietin-1 and -2 levels in coronary artery disease: Effects of angioplasty. Lip Ann. Med..

[60] Yueniwati Y., Darmiastini N., Arisetijono E. (2016). Thicker carotid intima-media thickness, and increased plasma VEGF levels suffered by post-acute thrombotic stroke patients. Int. J. Gen. Med..

[61] Sirico G., Spadera L., De Laurentis M., Brevetti G. (2009). Carotid artery disease and stroke in patients with peripheral arterial disease. The role of inflammation. Monaldi Arch. Chest Dis..

[62] Hashimoto H., Kitagawa K., Kuwabara K., Hougaku H., Ohtsuki T., Matsumoto M., Hori M. (2003). Circulating adhesion molecules are correlated with ultrasonic assessment of carotid plaques. Clin. Sci..

[63] Yi H., Li L., Wang Y., Tao H., Yu X., Yu B., Gao X., Lin P. (2022). The Potential Mediating Effects of Inflammation on the Association Between Type D Personality and Coronary Plaque Vulnerability in Patients With Coronary Artery Disease: An Optical Coherence Tomography Study. Psychosom. Med..

[64] Kabłak-Ziembicka A., Przewłocki T., Stępień E., Pieniążek P., Rzeźnik D., Sliwiak D., Komar M., Tracz W., Podolec P. (2011). Relationship between carotid intima-media thickness, cytokines, atherosclerosis extent and a two-year cardiovascular risk in patients with arteriosclerosis. Kardiol. Pol..

[65] Mizia-Stec K., Gasior Z., Zahorska-Markiewicz B., Janowska J., Szulc A., Jastrzebska-Maj E., Kobielusz-Gembala I. (2003). Serum tumour necrosis factor-alpha, interleukin-2 and interleukin-10 activation in stable angina and acute coronary syndromes. Comp. Study Coron. Artery Dis..

